# Effectiveness of a home fortification programme with multiple micronutrients on infant and young child development: a cluster-randomised trial in rural Bihar, India

**DOI:** 10.1017/S000711451800140X

**Published:** 2018-07-28

**Authors:** Leila M. Larson, Melissa F. Young, Patricia J. Bauer, Rukshan Mehta, Amy Webb Girard, Usha Ramakrishnan, Pankaj Verma, Indrajit Chaudhuri, Sridhar Srikantiah, Reynaldo Martorell

**Affiliations:** 1 Nutrition and Health Sciences Program, Laney Graduate School, Emory University, Atlanta, GA 30322, USA; 2 The Hubert Department of Global Health, Rollins School of Public Health, Emory University, Atlanta, GA 30322, USA; 3 Department of Psychology, Emory University, Atlanta, GA 30322, USA; 4 CARE India, 800013 Bihar, India

**Keywords:** Child development, Motor development, Mental development, Micronutrients, Multiple micronutrient powder

## Abstract

Research demonstrates the importance of nutrition for early brain development. Few studies have examined the effectiveness of multiple micronutrient powders (MNP) on child development. This study examined the impacts of home fortification with MNP on motor and mental development, executive function and memory of children living in Bihar. This two-arm cluster-randomised effectiveness trial selected seventy health sub-centres to receive either MNP and nutrition counselling (intervention) or nutrition counselling alone (control) for 12 months. Front-line health workers delivered the intervention to all households in study communities with a child aged 6–18 months. Data were collected using cross-sectional surveys at baseline and endline by selecting households from intervention (baseline, *n* 2184; endline, *n* 2170) and control (baseline, *n* 2176; endline, *n* 2122) communities using a two-stage cluster-randomised sampling strategy. Children in the intervention group had a significantly larger improvement from baseline to endline compared with those in the control group on scores for motor and mental development (Cohen’s *d*, motor=0·12; 95 % CI 0·03, 0·22; mental=0·15; 95 % CI 0·06, 0·25). Greater impacts of MNP on motor and mental development were observed in children from households with higher stimulation scores at baseline compared with those with lower stimulation (Cohen’s *d*, motor=0·20 *v*. 0·09; mental=0·22 *v*. 0·14; *P*_interaction_<0·05). No significant treatment differences were seen for executive function or memory. Home fortification with MNP through the existing health infrastructure in Bihar was effective in improving motor and mental development and should be considered in combination with other child development interventions such as stimulation.

Estimates indicate that 250 million children (43 %) under 5 years of age do not fulfil their developmental potential in low- and middle-income countries (LMIC)^(^ ^1^ ^)^. Poor development in early life has ongoing and long-term implications on school achievement and income and productivity (which tend to be lower), high fertility and poor care for the next generation^(^ ^2^ ^,^ ^3^ ^)^. Proper nutrition especially during the first 1000 d can influence early development directly through brain development, and indirectly through reducing illness and improving growth, as well as enhancing the child’s interactions with their environment^(^ ^4^ ^)^.

Recent meta-analyses and reviews document the efficacy of early child nutrition interventions on development especially among children under 2 years of age from LMIC^(^ ^5^ ^–^ ^7^ ^)^. Home fortification with multiple micronutrient powders (MNP) is a simple intervention that requires caregivers to mix a sachet of micronutrients into their child’s food before feeding. Although several studies have evaluated the efficacy of MNP on young child development, few have studied the effectiveness of such programmes when delivered using the existing government health infrastructure^(^ ^8^ ^–^ ^10^ ^)^. Evidence from effectiveness trials demonstrates the potential for such programmes to benefit at-risk populations with little additional investment and is important if state governments are to transition to scale at a regional level^(^ ^8^ ^)^.

Despite overall benefits of postnatal food and micronutrient interventions, trials have yielded inconsistent results on child development^(^ ^6^ ^)^. One reason could be the outcome measurement. Global measures of child development, such as the Bayley’s Scales of Infant and Child Development, which are typically used in nutrition studies in LMIC^(^ ^6^ ^)^, can capture effects on motor and mental development broadly. Because of nutrients’ specific functions in synaptic efficiency, myelination and hippocampal development^(^ ^11^ ^)^, micronutrient interventions may differentially affect specific cognitive functions that are masked in higher-order assessment tools. Two such specific processes that underlie early cognitive development^(^ ^12^ ^–^ ^14^ ^)^ and are hypothesised to be related to nutritional status are executive functions (cognitive processes associated with mental control and self-regulation)^(^ ^15^ ^,^ ^16^ ^)^ and memory^(^ ^17^ ^–^ ^19^ ^)^.

The objectives of the current study were to examine the effectiveness of home fortification with MNP on motor and mental development of children aged 6–18 months, and on executive function and declarative memory of children aged 12–18 months. We hypothesised that children from communities receiving the intervention would score higher on development, executive function and memory tests than children from control communities. Our study was a cluster-randomised effectiveness trial conducted in rural India, a country that contributes a quarter of all children at risk of poor development worldwide^(^ ^20^ ^)^. The trial was designed based on a request from the government of Bihar for further evidence to address micronutrient deficiencies and their consequences in this population. Although MNP have previously been used in India^(^ ^21^ ^,^ ^22^ ^)^, to our knowledge no large-scale effectiveness trials have examined the potential impact of such a programme using the existing front-line health workers (FLW) on early child development in this context.

## Methods

This study was a collaboration between CARE India and Emory University. It was approved by the Institutional Review Boards of the 3rd Futures Group, Delhi, India, St John’s Medical College & Hospital Institutional Ethics Committee, Bangalore, India, and Emory University, Atlanta, USA, and registered with the US National Institute of Health as a clinical trial (www.ClinicalTrials.gov; NCT02593136).

### Setting

The government of Bihar, in partnership with the Bill and Melinda Gates Foundation and CARE India, launched the Integrated Family Health Initiative (IFHI) in 2011 to address pressing health challenges faced by its population. The IFHI identified childhood anaemia (at 64 % in children under 5 years in Bihar)^(^ ^23^ ^)^ as one of the major public health priorities to be targeted. On the basis of evidence^(^ ^24^ ^)^, home fortification with MNP was identified as an innovative strategy to address this priority. The programme was delivered by government FLW with the support of CARE India, the primary implementing partner of IFHI and Emory University that provided nutrition technical support. The goal of the study was to determine the effectiveness of the programme in one district of Bihar before potential state-wide scale-up.

Extensive formative research was conducted between 2012 and 2014 to establish viability of the MNP product in this context (e.g. acceptability of the product, messaging and visual messaging)^(^ ^25^ ^)^. The current study was started following positive evidence for its acceptability by the community.

### Study design and participants

**Fig. 1:**
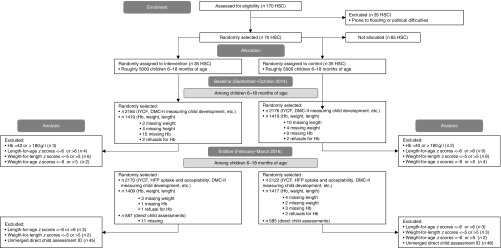
CONSORT flow diagram. Owing to families being out-of-home during the harvest and festival season, which coincided with our endline, forty-eight households were under-sampled for the household survey (total 4292 at endline). At baseline and endline, 1 % of children were oversampled for anthropometry (total 2838 at baseline; 2826 at endline). At endline, eleven households were missing direct child assessments owing to sickness (total 1172). DMC-II, Developmental Milestones Checklist II; IYCF, infant and young child feeding; HSC, health sub-centre; HFP, home fortification project.

We conducted a 12-month two-arm cluster-randomised effectiveness trial in West Champaran district of Bihar, India, between January 2015 and December 2015 (Fig. 1). In India, districts are subdivided into blocks (subdistricts). Four blocks were purposefully chosen to include two blocks close to and two blocks far from district headquarters. Within each of these four blocks, health sub-centres (HSC) that were prone to flooding and political difficulties (*n* 35) were excluded. Out of 135 HSC communities, seventy were randomly assigned, using a simple randomisation method with random number generator, to intervention or control communities (Fig. 1); all families with children aged 6–18 months in these communities received complementary feeding counselling, and intervention families additionally received the home fortification product, a MNP labelled Jeevan Jyoti (the name means ‘the light of life’ in Hindi, chosen by community members as part of formative research).

We utilised a nested cross-sectional pre-test–post-test control group design^(^ ^26^ ^)^ whereby two cross-sectional surveys of children aged 6–18 months were used to assess the change in outcomes. This design is appropriate for evaluations of universal programmes. The baseline (*n* 4360) and endline (*n* 4292) surveys were conducted from August to September 2014 and from February to March 2016, respectively. Inclusion criteria for survey enrolment were child’s age above 6 months and below 18 months, half of whom were 6–11·9 months of age and the other half were 12–18 months of age. Cross-sectional surveys of children aged 6–18 months at baseline and endline were chosen in order to stay as true to an effectiveness study as possible and to examine children who were consuming complementary foods and therefore would have the potential to benefit from MNP.

For the baseline and endline evaluation, a household listing (list of eligible households) was performed before starting the survey. In HSC with five or more Anganwadi centres (AWC), simple random sampling (SRS) was used to select five AWC for which household listing was performed. Using the household listings, thirty-one children aged 6–11·9 months and thirty-one children aged 12–17·9 months from each HSC were chosen using SRS to be included in the household survey, which included questions on their child’s mental and motor development. Refusals for the household survey were replaced (*n* 2 at baseline). From each age group in each HSC, twenty of the thirty-one children were randomly selected to be measured for anthropometry. At endline only, seventeen children in each HSC from the older age group who received the household survey and anthropometry were chosen using SRS to be tested using direct child assessments, through game-like interactions with each child (refer to the ‘Measurements’ section). Because additional funding was obtained after the baseline was completed, direct child assessments were performed only at endline (Fig. 1).

During the survey, supervisors conducted 10 % back-checks that involved re-interviewing the caregiver on a random subset of questions and comparing results with the field investigators’ results, and 10 % spot-checks that involved observing interviews.

### Intervention

The counselling and Jeevan Jyoti MNP were delivered to households with a child aged 6–18 months at no cost to the families, by the local community FLW, the Accredited Social Health Activists (ASHA) and the Anganwadi Workers (AWW). ASHA and AWW coordinated so that they each visited approximately half the households in their catchment area. ASHA are local women trained as health educators under the Ministry of Health. AWW are part of the Integrated Child Development Services programme in India; in addition to family planning and nutrition counselling and supplementation, they administer preschool activities for children aged 3–5 years. Each HSC is home to approximately seven ASHA and seven AWW. Typically, a pair of one AWW and one ASHA work within one AWC catchment area. The project area included a total of roughly 10 000 children.

FLW were advised to provide all households (intervention and control communities) with counselling and information pamphlets on infant and young child feeding (IYCF) practices that included guidance on breast-feeding, food variety, frequency of feeding, food consistency, food quantity, hand washing and hygiene practices.

In intervention communities, FLW distributed a box of 30 MNP sachets to the caregivers of children aged 6–18 months on a monthly basis and provided instructions to mix one MNP sachet into the child’s food every day. Each MNP sachet contained 12·5 mg of Fe as ferrous fumarate, 5 mg of zinc gluconate, 0·16 mg of folic acid, 0·3 mg of vitamin A acetate, 30 mg of ascorbic acid, 0·9 µg of vitamin B_12_ and 90 µg of iodine (roughly one Recommended Dietary Allowance^(^ ^27^ ^)^ for most nutrients). Children aged in and out of the programme during its 1-year duration. FLW were advised to provide four boxes of MNP to children during each of the time periods of 6–12 and 12–18 months in accordance with World Health Organization recommendations^(^ ^24^ ^)^. In intervention communities, the IYCF pamphlet also included detailed graphic instructions on the use of MNP. At the start of the programme, and on a monthly basis over the project period, meetings and refresher trainings were held at the HSC level between CARE project staff and FLW to go over the programme, the proper use of MNP sachets and their distribution, the IYCF counselling and collection of monthly monitoring data.

### Blinding and training

Both data collection and data entry were blinded to the intervention. Data collectors were present in the communities only before and after the intervention.

The data collection team comprised supervisors, household survey data collectors, anthropometry/Hb data collectors and research assistants for the direct child assessments. All had received minimal information on the nutrition programme and were unaware of the households’ status. All had at least completed secondary school. Local research assistants conducting direct child assessments were bachelors, masters or PhD students in Psychology or Social Science. All data collectors and research assistants were trained in their respective work over a 2-week period. The questionnaire and direct child assessment tasks were pilot tested on children from Bihar and adapted before starting the survey. Training on direct child assessments was performed by L. M. L. and a local psychologist. Ongoing monitoring ensured adherence to study protocols.

### Ethical considerations

Written informed consent (or thumb print from participants unable to write their name) was obtained from all caregivers participating in the survey. Participants were informed that the decision to participate was entirely theirs and that, if they chose to participate, they could withdraw at any time. Refusal to participate in the survey did not exclude them from receiving the MNP or other services from the FLW they would normally receive. Consent for child anthropometry, Hb and direct child development assessments was obtained separately, as these data were obtained in a subset of the children; caregivers could opt out of having these measurements, and still be included in the general components of the survey.

### Measurements

All questionnaires and tasks were administered in Hindi or Bhojpuri, the local dialect, based on the fluency of the respondent. The questionnaire was translated and back-translated to ensure that the language was correct. Data collectors read all questions out loud to mothers or caregivers.

#### Developmental milestones

Primary outcomes were motor (gross and fine) and mental (language and personal–social) development, assessed at baseline and endline using the Developmental Milestones Checklist II (DMC-II), a seventy-five-item parent report^(^ ^28^ ^)^. A subset of the DMC along with seven cognitive items showed convergent validity against the Bayley Scales of Infant and Toddler Development^(^ ^29^ ^)^. Motor development includes the sum of scores from the gross and fine motor subscales; mental development includes the sum of scores from the language, personal–social and cognitive subscales. Items were scored as 1 if the child had performed this activity and 0 if the child had not yet performed it. This measure has been validated in India, Burkina Faso, Kenya and Ghana^(^ ^28^ ^,^ ^30^ ^–^ ^32^ ^)^.

#### Executive function

Executive function was a secondary outcome measured at endline only in children aged 12–18 months using a direct child assessment. Executive function and memory (as described below) were measured in children aged 12–18 months only because we wanted to examine these specific functions in children who would have had the opportunity to consume the MNP for a longer period of time. Research assistants worked in pairs; one interacted with the child, whereas the other took notes and scored the measure. In the A-not-B task^(^ ^33^ ^)^, the child is shown a desirable object hidden under a cloth (location A), within the child’s reach. After a brief delay, the child is allowed to search for and find the object. After several successes finding the object at a particular location, the object is then hidden under a cloth at an alternate location (B). Here, correct performance depends on the child’s ability to update their memory of the hiding place, as well as to inhibit the response of searching at the location where the object was previously found. Each child was given four trial attempts to retrieve the object successfully under a given delay. If the child was successful in retrieving the object on two consecutive trials, the side of hiding was changed and the delay incremented by 3 s. This was continued until the child failed to retrieve the object on two consecutive trials or the maximum of 12-s delay was successfully passed (delays: 0, 3, 6, 9 and 12 s) (online Supplementary Fig. S1). Further details on the test are described in the online Supplementary Table S1.

#### Memory test

Memory was another secondary outcome measured at endline only in children aged 12–18 months. In the Elicited Imitation task^(^ ^34^ ^,^ ^35^ ^)^, which measures episodic memory, the child was tested for immediate and delayed recall of two 2-step tasks and two 3-step sequences of action. The research assistant, sitting in front of the child, modelled and narrated the sequence of actions in succession two times. They then returned the props to the child and invited them to imitate the exact sequence. Each child was tested on one 2-sequence task and one 3-sequence task immediately after modelling and one 2-sequence task and one 3-sequence task after a delay of 10 min (different tasks to those used for immediate recall). A 10-min delay is long enough to reveal a deficit in individuals with compromised memory function owing to medial–temporal lobe damage^(^ ^36^ ^)^, and thus it is especially sensitive to the developmental integrity of the hippocampus^(^ ^34^ ^)^. To score the children’s behaviour, for each sequence, we calculated a total number of individual target actions produced (maximum=2 for 2-sequence tasks, maximum=3 for 3-sequence tasks) and the total number of pairs of actions produced in the target order (maximum=1 for 2-sequence tasks, maximum=2 for 3-sequence tasks). Only the first occurrence of each target action was considered so as to reduce credit that might be received owing to chance or trial and error^(^ ^34^ ^)^. Further details on the test are described in the online Supplementary Table S1.

#### Stimulation, diet and morbidity

The Family Care Indicators (FCI), a nine-item parent–report measure, previously validated in South Asia^(^ ^37^ ^)^, was used to assess stimulating caregiving. The FCI includes questions on play materials, spending time outside the home with the child and reading, telling stories, singing, playing and naming and counting with the child. A Wealth Index, using five categories, was calculated using Principal Component Analysis with family assets, type of household, land ownership and source of drinking water; this was done separately for baseline and endline data. A child dietary diversity score, minimum meal frequency value and minimum acceptable diet value were created according to World Health Organization guidelines^(^ ^38^ ^)^. Food deprivation was assessed through mothers’ report using the cross-culturally validated Household Hunger Scale^(^ ^39^ ^)^. Households were classified as food deprived or not based on their responses to the four-item Likert scale. Recent morbidity was measured as any fever, cough or diarrhoea in the past 2 weeks reported by the caregiver. A total of thirty-two field investigators, trained and standardised, collected the household survey information, including the DMC-II, at baseline and endline.

#### Anthropometry and Hb

Anthropometric measurements including weight, length and mid-upper arm circumference (MUAC) were taken at baseline and endline following standard procedures^(^ ^40^ ^)^. Weight was assessed with the Seca 874 (Seca) and length with the Seca 417. Length-for-age, weight-for-length and weight-for-age *z* scores were calculated using the WHO 2006 child growth standards^(^ ^40^ ^)^; *z* scores <−2 were used to define stunting, wasting and underweight, respectively; *z* scores <−3 were used to define severe stunting, severe wasting and severe underweight. MUAC tapes (S0145620 MUAC, Child 11.5 Red/PAC-50) were used to measure MUAC. Hb was measured with the HemoCue Hb 201+ Analyzar (HemoCue). Blood samples were taken using a heel prick from children aged 6–11 months, and a finger prick for children aged 12–18 months. Child anaemia was defined as mild if 100 g/l≤Hb<110 g/l, moderate if 70 g/l≤Hb<100 g/l and severe if Hb <70 g/l^(^ ^41^ ^)^. If children were found to be severely anaemic, they were referred to the nearest primary health centre. Effects on anaemia and stunting have been reported elsewhere^(^ ^42^ ^)^.

### Sample size estimation

For the primary outcome of mental and motor development using the DMC-II, a sample size of 2170 children per group across seventy clusters was calculated to detect an effect size of 0·1 or larger, 80 % power, *α* of 0·05 and an intra-cluster correlation coefficient of 0·01. For the secondary outcomes of executive function and memory, a sample size of 546 per group across seventy clusters was calculated to detect an effect size of 0·25 or larger, assuming a power of 0·8, an *α* of 0·05, an intra-cluster correlation coefficient of 0·2 and a refusal rate of 5 %. These estimates were based on the literature on executive function and memory, and related child development interventions^(^ ^43^ ^–^ ^46^ ^)^.

### Statistical analyses

Data were analysed using SAS 9.4 (SAS Institute). Children with Hb measurements below 40 g/l or above or equal to 180 g/l^(^ ^47^ ^)^ (0·2 % at baseline, 0·01 % at endline), or children with length-for-age *z* scores <−6 or >6 (0·2 % at baseline, 0·2 % at endline), weight-for-length *z* scores <−5 or >5 (0·6 % at baseline, 0·02 % at endline) or weight-for-age *z* scores <−6 or >5 (0·5 % at baseline, 0·01 % at endline) were omitted from the analysis (Fig. 1)^(^ ^48^ ^)^.

The effect of the intervention on change in DMC-II scores (mental and motor development, and all subscales separately) was identified by linear mixed effects models with a fixed effect of intervention group and a random effect of HSC cluster with nested AWC. We used a difference-in-difference approach to run intent-to-treat analyses (model 1) using intervention group, survey (baseline *v*. endline), their interaction and age of the child in months as the only fixed effects. Model 2 used the same fixed effects as Model 1, in addition to covariates that were significantly different between the intervention and control communities at baseline (baseline Hb and baseline household stimulation score, wealth index, maternal education, caste and young mother status). This study used two cross-sectional surveys, and did not follow-up the same children; therefore, in order to impute a baseline Hb and baseline household stimulation score to endline children, we used the mean of the Hb concentration and household stimulation score for children in their same cluster, stratified by sex and age group. The interaction was tested by using the standard error and denominator df that reflected the HSC level with nested AWC. Cohen’s *d* effect sizes are reported for each outcome.

Effects of the intervention on executive function and memory outcomes were examined using linear and generalised linear mixed models with a fixed effect of intervention group and random effect of HSC cluster with nested AWC. Outcomes of interest on the A-not-B task were: (1) ability to tolerate either 3, 6, 9 or 12 s, or not tolerating any delay; (2) the ability to find the object under cloth A; and (3) the ability to find the object under cloth B. Outcomes from the Elicited Imitation tasks included: (1) number of actions completed in all sequences (continuous) and (2) number of pairs of actions completed in the correct order in all sequences (continuous). All sequences were summed because there was no significant difference in the outcomes for sequences performed with or without the 10-min delay. Performance on the tasks during the time recorded as ‘baseline’ (pre-demonstration) was significantly different from that recorded post demonstration, indicating that the actions performed post demonstration were due, as intended, to memory. Model 1 was intent to treat adjusting for age of the child in months, and model 2 adjusted for the same covariates as those described for model 2 with the DMC-II scores, in addition to child baseline mental development scores using the DMC-II, as well as the research assistant testing the child. Concurrent validity of the A-not-B and Elicited Imitation tasks was examined by the associations between outcomes for each test and age of the child. Cohen’s *d* effect sizes were calculated by taking a difference of the change in scores between groups (or difference in scores between groups for measurements only conducted at endline) divided by a pooled standard deviation.

Using model 2, we examined effect modification on mental and motor development scores using the DMC-II and the outcomes of the A-not-B and Elicited Imitation tasks, from level of baseline household FCI stimulation (dichotomised as low *v*. high based on a score below/equal to or above the median score of 5) and continuous baseline Hb concentrations. We also examined dose–response within the intervention group using number of MNP sachets consumed by the child in the past month (dichotomised as <10 *v*. ≥10 sachets in children from intervention communities at endline only). Baseline household FCI stimulation scores and baseline Hb concentrations were imputed for endline children by taking a mean of the FCI score or Hb concentration within baseline children from the same HSC of the same sex and age group. There was little (7 %) overlap between children with high baseline stimulation scores and children having consumed ≥10 MNP sachets over the past month, indicating that the two measures are examining different outcomes. Statistical significance was defined as *P* value<0·05.

### Quality control

Reliability of the data collectors’ scores was established by examining the relation between the field investigators’ scores and those of an expert when assessing the same child, using five children, both at baseline and endline^(^ ^40^ ^)^. Reliability measurements for weight, height and MUAC yielded a Pearson’s correlation coefficient between measurements of the investigators and expert of >0·92 and a coefficient of reliability (which measures the proportion of inter-subject variance due to measurement error) of >0·80. Reliability of the research assistants on direct child assessments was difficult to establish given the tasks tested executive function and memory; any re-test would have yielded biased responses from the child. Therefore, all research assistants practiced the tasks on children for 3 d while being observed by the trainers. Only those who performed the assessments correctly, as determined by the first author and a local psychologist, were retained. A field coordinator and supervisor monitored assessments throughout data collection to ensure fidelity to the protocols.

Intra-cluster correlation coefficients for each outcome were calculated using the between- and within-cluster variability: <0·01 for motor, 0·02 for mental, <0·01 for gross motor, 0·03 for fine motor, 0·02 for language, 0·03 for personal–social, 0·05 for cognitive scores, 0·10 for memory scores and 0·07 for executive function scores.

## Results

### Baseline characteristics of children

At baseline, 60 % of mothers had no schooling, mean maternal age was 25·2 years, mean parity was 2·7 children, 72 % of children were anaemic, 33 % were stunted, 27 % were wasted and 42 % were underweight. At baseline, the mean motor and mental development score among all children was 17·8 out of 32 (95 % CI 17·6, 18·0) and 24·9 out of 50 (95 % CI 24·4, 25·3), respectively. The intervention and control groups matched on most key demographic and child nutritional characteristics at baseline (Tables 1 and 2). However, the intervention group had significantly higher prevalence of any maternal education, higher mean FCI score, lower mean Hb concentration and higher mean score on the cognitive subscale of the DMC-II. Among children living in intervention communities at endline, 40 % received ten or more MNP sachets over the month before the survey.

**Table 1:** Household characteristics at baseline and endline for the multiple micronutrient powders (MNP) intervention group and the control group* (Percentages and numbers; mean values and standard deviations)

	Baseline				Endline			
	Intervention group (*n* 2184)		Control group (*n* 2176)		Intervention group (*n* 2170)		Control group (*n* 2122)	
	%	*n*	%	*n*	%	*n*	%	*n*
Religion								
Hindu	78·1	1701	79·5	1730	75·7	1646	78	1655
Muslim	21·9	478	20·5	445	24·3	529	22	466
Maternal age (years)								
Mean	25·2		25·2		25·0		25·2	
sd	4·7		4·8		4·7		4·7	
Paternal age (years)								
Mean	29·5		29·4		28·9		29·3	
sd	5·7		5·9		5·3		5·5	
Maternal education								
Any schooling	42·1	918	37·7	819	48·0	1043	44·9	952
Paternal education								
Any schooling	65·1	1417	62·8	1360	68·2	1453	67·4	1397
Caste (self-reported)								
Scheduled caste	24·1	524	26·4	572	18·1	394	21	446
Scheduled tribe	7·3	159	9·3	201	7·2	156	11·3	241
Other backwards caste	17·0	370	15	325	13·7	298	11	234
Parity								
Mean	2·7		2·7		2·6		2·7	
sd	1·6		1·6		1·6		1·6	
Family Care Indicators score (out of 13)								
Mean	5·2		5		5·3		5·2	
sd	2·3		2·3		1·7		1·5	

* At baseline, total *n* 4360 for all measurements. At endline, total *n* 4292 for all measurements. The self-reported castes are officially designated groups in India.

**Table 2:** Demographic and clinical characteristics of children at baseline and endline for the multiple micronutrient powders (MNP) intervention group and the control group* (Percentages and numbers; mean values and standard deviations)

	Baseline				Endline			
	Intervention group (*n* 2184)		Control group (*n* 2176)		Intervention group (*n* 2170)		Control group (*n* 2122)	
	%	*n*	%	*n*	%	*n*	%	*n*
Age (months)								
6–11	50·2	1096	51·1	1111	46·9	1021	46·3	983
12–18	49·8	1087	48·9	1065	53·1	1154	53·7	1141
Age in months in the 6–11-month age group								
Mean	8·6		8·7		8·4		8·4	
sd	1·7		1·7		1·7		1·7	
Age in months in the 12–18-month age group								
Mean	14·1		13·9		14·6		14·6	
sd	1·8		1·8		1·7		1·7	
Sex								
Girls	48·7	1062	50·8	1103	47·2	1026	46·6	990
Dietary diversity score (out of 7)								
Mean	2·7		2·8		3·6		3·6	
sd	1·1		1·1		1·6		1·6	
Minimum meal frequency achieved	64·5	1380	67·1	1428	71·8	1418	75·1	1451
Minimum acceptable diet achieved	14·4	314	16	348	22·3	486	23·1	491
Any household hunger	9·7	209	9·3	201	4·3	93	4·6	97
Any recent child morbidity	75·2	1642	76·2	1658	51·7	1125	54·4	1157
Fever	64·8	1415	67	1459	34·7	755	37·6	799
Cough	54·3	1184	55·5	1205	39·5	859	39·4	837
Diarrhoea	11·6	253	12·1	263	11·1	241	15·6	331
Anthropometry								
Length-for-age *z* score								
Mean	−1·49		−1·49		−1·36		−1·38	
sd	1·3		1·3		1·2		1·2	
Weight-for-length *z* score								
Mean	−1·34		−1·35		−0·83		−0·91	
sd	1·1		1·2		1		1	
Weight-for-age *z* score								
Mean	−1·80		−1·82		−1·32		−1·40	
sd	1·1		1·1		1·1		1·1	
Mid-upper arm circumference								
Mean	13·1		13·1		13·6		13·6	
sd	1·1		1·1		1		1	
Anaemia								
Any (Hb<110 g/l)	75·3	1062	69·2	977	64·4	910	66·1	934
Mild (100 g/l≤Hb<110 g/l)	27·5	388	28·4	401	30·8	435	32	453
Moderate (70 g/l≤Hb<100 g/l)	44·8	632	38·5	543	32·4	458	33·5	473
Severe (Hb<70 g/l)	3·0	42	2·3	33	1·2	17	0·6	8
Hb (g/l)								
Mean	99		102		104		104	
sd	15		16		14		14	

* At baseline, total *n* 4360 for all measurements except for anthropometry and anaemia, for which *n* 2838. At endline, total *n* 4292 for all measurements except for anthropometry and anaemia, for which *n* 2826. Minimum meal frequency is achieved if a breast-fed child is fed at least three times or a non-breast-fed child is fed at least four times the previous day. Minimum acceptable diet is achieved if a breast-fed child is fed four or more food groups and achieved minimum meal frequency or a non-breast-fed child received at least two milk feeds, is fed four or more foods groups and achieved minimum meal frequency the previous day.

### Concurrent validity of child development measures

Validity of the DMC-II in this context is reported elsewhere^(^ ^31^ ^)^. In a bivariate regression, outcomes on the A-not-B and Elicited Imitation tasks are significantly (*P*<0·001) associated with child age. Each month of age between 12 and 18 months was associated with a 13 % (OR 1·13; 95 % CI 1·06, 1·20) higher odds of tolerating three more seconds of delay before finding a hidden object in the A-not-B task, a 0·28 (95 % CI 0·22, 0·34) increase in the number of actions complete in the correct order across all Elicited Imitation sequences and a 0·19 (95 % CI 0·15, 0,24) increase in the number of pairs of actions complete in the correct order. For the Elicited Imitation tasks, children’s baseline performance (scores for actions completed before research assistant demonstrating the sequences) was significantly different from their post-demonstration performance for each sequence, meaning that performance was owing to memory and not spontaneous production of actions or sequences of action. These patterns are consistent with those in the wider literature using Elicited Imitation tasks^(^ ^34^ ^)^.

### Impact of multiple micronutrient powder intervention on motor and mental development

DMC-II scores for mental and motor development and for each subscale, as well as Elicited Imitation task outcomes, were normally distributed. The difference-in-difference analyses for DMC-II scores indicated that scores of children from intervention communities increased significantly more than those of control communities in gross motor, language and personal–social development subscales (*P*<0·01) (Table 3). As a whole, the change in motor and mental development of children between the two time points was larger for the intervention group children than the control group children (Cohen’s *d* effect size for motor=0·12 (95 % CI 0·03, 0·22); for mental=0·15 (95 % CI 0·06, 0·25)) (online Supplementary Fig. S2 and S3). Examination by age showed that the change in scores for gross motor development was significantly larger in the intervention group than in the control group for children aged 6–11 months, whereas the change in language development scores was significantly larger for children aged 12–18 months. Significant effect modification was seen by level of baseline household stimulation, but not by baseline Hb. The effect of the intervention on motor and mental development, and language, and personal–social subscales, in children from households with high levels of stimulation was significantly more than that of children with low levels of household stimulation (online Supplementary Table S2). A significant dose–response was observed using numbers of MNP sachets consumed by the child over the previous month. At endline, among children from the intervention communities, scores for motor and mental development and each subscale (gross and fine motor, language, personal–social and cognitive development) were significantly higher in children who had received ten or more MNP sachets over the previous month compared with children who had received fewer sachets (online Supplementary Table S2).

**Table 3:** Developmental Milestones Checklist II (DMC-II) scores for children aged 6–18 months at baseline and endline for the multiple micronutrient powders (MNP) intervention group and the control group* (Mean values and standard deviations; adjusted means and 95 % confidence intervals)

	Baseline				Endline									
	Intervention (*n* 2183)		Control (*n* 2176)		Intervention (*n* 2170)		Control (*n* 2122)		Model 1			Model 2		
	Mean	sd	Mean	sd	Mean	sd	Mean	sd	Effect size (mean)	95 % CI	*P*	Effect size (mean)	95 % CI	*P*
All children														
Motor development	17·8	5·4	17·9	5·3	18·8	6·6	18·5	6·4	0·13	0·05, 0·21	0·003	0·12	0·03, 0·22	0·012
Mental development	24·9	6·8	24·8	6·8	26·2	7·5	25·4	7·5	0·16	0·07, 0·24	<0·001	0·15	0·06, 0·25	0·002
Gross motor	11·1	4·0	11·1	4·0	12·1	4·9	11·9	4·7	0·15	0·07, 0·23	0·001	0·15	0·06, 0·25	0·002
Fine motor	6·7	1·9	6·7	1·9	6·6	2·1	6·6	2·0	0·04	−0·04, 0·13	0·314	0·02	−0·08, 0·12	0·685
Language	4·6	2·5	4·6	2·5	5·4	2·7	5·1	2·6	0·16	0·07, 0·24	<0·001	0·17	0·07, 0·26	0·001
Personal–social	16·1	3·7	16·2	3·7	16·5	4·1	16·2	4·0	0·14	0·06, 0·23	0·001	0·13	0·04, 0·23	0·006
Cognitive	4·2	1·6	4·1	1·6	4·3	1·7	4·1	1·7	0·07	−0·02, 0·15	0·130	0·05	−0·04, 0·15	0·293
Children aged 6–11 months														
Motor development	14·5	4·0	15·0	4·2	13·7	4·7	13·6	4·6	0·14	0·02, 0·26	0·023	0·16	0·02, 0·29	0·024
Mental development	21·6	5·8	21·7	5·9	21·1	6·5	20·3	6·3	0·14	0·02, 0·26	0·022	0·14	0·01, 0·28	0·041
Gross motor	8·6	2·6	9·0	2·9	8·3	3·2	8·2	3·1	0·16	0·04, 0·28	0·011	0·19	0·05, 0·32	0·008
Fine motor	5·9	1·9	6·0	1·9	5·4	2·0	5·3	1·9	0·07	−0·05, 0·19	0·269	0·06	−0·07, 0·20	0·366
Language	3·4	2·0	3·5	1·9	3·6	2·0	3·4	1·9	0·08	−0·04, 0·21	0·171	0·09	−0·05, 0·23	0·191
Personal−social	14·5	3·4	14·7	3·5	13·9	3·7	13·6	3·6	0·14	0·02, 0·26	0·026	0·13	−0·01, 0·27	0·067
Cognitive	3·7	1·6	3·6	1·6	3·6	1·8	3·3	1·8	0·11	−0·01, 0·23	0·073	0·11	−0·02, 0·25	0·109
Children aged 12–18 months														
Motor development	21·1	4·5	20·9	4·5	23·2	4·4	22·7	4·4	0·10	−0·02, 0·22	0·088	0·08	−0·06, 0·21	0·260
Mental development	28·3	6·0	28·1	6·1	30·7	5·1	29·8	5·3	0·16	0·04, 0·28	0·009	0·16	0·02, 0·29	0·021
Gross motor	13·6	3·6	13·4	3·7	15·5	3·5	15·0	3·5	0·13	0·02, 0·25	0·026	0·12	−0·02, 0·25	0·086
Fine motor	7·5	1·5	7·5	1·5	7·7	1·5	7·7	1·4	−0·01	−0·13, 0·11	0·883	−0·05	−0·18, 0·09	0·486
Language	5·8	2·4	5·8	2·5	6·9	2·1	6·5	2·2	0·21	0·09, 0·33	0·001	0·22	0·08, 0·35	0·002
Personal–social	17·8	3·3	17·7	3·3	18·8	2·8	18·4	2·9	0·13	0·01, 0·25	0·031	0·13	0·00, 0·26	0·057
Cognitive	4·7	1·4	4·5	1·5	5·0	1·3	4·8	1·3	0·00	−0·12, 0·12	0·970	−0·02	−0·15, 0·11	0·778

* All models account for clustering by health sub-centre and nested Anganwadi Centre. Model 1 is adjusting for age of the child. Model 2 is adjusting for the age of the child, baseline Hb, baseline home stimulation score, wealth index, maternal education, caste and young mother. Effect sizes are calculated with adjusted means. Motor development scale ranges from 0 to 32, mental development from 0 to 50, gross motor from 0 to 22, fine motor from 0 to 10, language from 0 to 15, personal–social from 0 to 28 and cognitive from 0 to 7. Children aged 6–11 months, *n* 2208 at baseline and *n* 2004 at endline; children aged 12–18 months, *n* 2152 at baseline and *n* 2288 at endline.

### Impact of multiple micronutrient powders intervention on executive function and memory

In children aged 12–18 months, there was no significant impact of the intervention on executive function outcomes. In the A-not-B task, there were no differences in the odds of tolerating a 3-, 6-, 9- and 12-s delay compared with no delay for the MNP intervention group compared with the control group (Table 4). We grouped all children who tolerated any delay because a small proportion (22 %) of children had a maximum tolerated delay between 0 and 12 s (i.e. a maximum tolerated delay of 3, 6 or 9 s). The majority of children who were able to tolerate any delay were able to tolerate a 12-s delay. There were no significant effects of the intervention on the odds of finding the toy under cloth A or the odds of perseverative error (not finding the toy under cloth B) (Table 4). Similarly, there was no effect of the intervention on memory scores using the Elicited Imitation task, whereby mean number of actions and pairs of actions complete in the correct order were not significantly different for the MNP intervention group compared with the control group (Table 4). There was no significant effect modification of the intervention from level of baseline household stimulation, baseline Hb nor was there a dose–response.

**Table 4:** Executive function outcomes and mean scores on Elicited Imitation memory tasks for multiple micronutrient powders (MNP) intervention group and control group among children aged 12–18 months (*n* 1078)* (Percentages and numbers; odds ratios and 95 % confidence intervals; mean values and standard deviations)

	Intervention		Control		Model 1			Model 2		
	%	*n*	%	*n*	OR	95 % CI	*P*	OR	95 % CI	*P*
Executive function										
Tolerated delay										
None	42·8	248	37·3	215	Ref.			Ref.		
Any	57·2	332	62·7	362	0·82	0·62, 1·1	0·27	0·79	0·59, 1·05	0·10
Not found under cloth A	26·1	153	22·9	134	Ref.			Ref.		
Found under cloth A	73·9	434	77·1	451	0·85	0·57, 1·25	0·33	0·80	0·55, 1·15	0·22
Perseverative error	32·2	189	28·2	165	Ref.			Ref.		
Found under cloth B	67·8	398	71·8	420	0·83	0·60, 1·16	0·23	0·82	0·59, 1·14	0·23
	Mean	sd	Mean	sd			*P*			*P*
Memory										
Number of actions complete in all paradigms	4·70	2·20	5·00	2·00	–		0·43	–		0·13
Number of pairs of actions complete in the correct order in all paradigms	1·70	1·30	1·90	1·20	–		0·76	–		0·38

Ref., referent value.

* All models account for clustering by health sub-centre and nested Anganwadi Centre. Model 1 is adjusting for age of the child. Model 2 is adjusting for baseline mental development scores using Developmental Milestones Checklist II, baseline Hb, baseline home stimulation score, age of the child, examiner, wealth index, maternal education, caste and young mother. Any tolerated delay includes 3-, 6-, 9- and 12-s delay.

## Discussion

This study was an effectiveness trial of home fortification with MNP, distributed through the existing health infrastructure in rural Bihar. Our findings indicate that the intervention had an impact on gross motor, language and personal–social development of children aged 6–18 months as measured by the DMC-II. The impact on motor development was significant in younger children aged 6–11 months, and the impact on language development was significant in older children aged 12–18 months. Effects were modified by the baseline household stimulation, whereby the intervention had a larger impact in children with higher compared with lower stimulation at the start of the intervention. We observed no significant effect of the intervention on executive function or memory of children aged 12–18 months.

The motor and language development effect sizes we observed in our study are smaller than those from a previous effectiveness trial in children aged 6–24 months in Pakistan in which MNP were delivered by Lady Health Workers^(^ ^7^ ^)^. However, in the Pakistan study, mothers of children under 2 years were given MNP and nutrition education over a period of 18 months, which is longer than the current study. Another effectiveness trial in Bangladesh utilised staff from a community health and development programme and provided iron and folic acid supplements during monthly home visits to mothers during pregnancy until 3 months postpartum and a 15-nutrient MNP to the child from 6 to 24 months of age^(^ ^9^ ^)^. The trial found a significant impact on motor and language development, but no impact on personal–social development^(^ ^9^ ^)^.

In younger children aged 6–11 months, significant improvements were observed in the gross motor subscale from baseline to endline in the intervention group compared with the control group, whereas in children aged 12–18 months significant improvements were observed in the language development subscale. These findings suggest that infants’ gross motor abilities were sensitive to the MNP supplementation from an early age, which may be because motor skills change more rapidly in the 1st year rather than the 2nd year of life^(^ ^49^ ^–^ ^51^ ^)^. Even though younger children would have received MNP for a shorter time period than older children, there is potential to benefit gross motor skills. Gross motor development itself has value for a young child in terms of increasing mobility; enhanced mobility can increase mental development especially if it raises the amount of environmental stimulation accessed by the child. The benefit to language development seen only in children after 12 months may be owing to MNP contributing to the ‘language explosion’ seen in the 2nd and 3rd year of life^(^ ^52^ ^)^ or to mothers’ greater awareness of their child’s language development owing to enhancement of children’s overt speech at this age. Previous research has shown a similar impact of nutrition on expressive language in children above 1 year of age. A recent cluster-randomised trial in Bangladesh, which provided MNP to children between 7 and 12 months of age and followed them up at 16–22 months of age, found improvements in expressive, but not receptive, language development^(^ ^10^ ^)^. Standard deviations of scores are similar across both age groups in our sample, showing that children are not reaching a ceiling or floor score at the tail ends of the age range. The score was able to capture variability from 6 to 18 months of age. It is worth noting that many children in this population begin complementary feeding later than recommended^(^ ^31^ ^)^; only children who had begun complementary feeding would have been exposed to the MNP, which were designed to be mixed into the child’s food.

Similarly to other recent nutrition interventions^(^ ^9^ ^,^ ^53^ ^–^ ^55^ ^)^, we did not find significant effects of the intervention on executive function or memory. The measures of executive function and memory could be insensitive to an MNP intervention in this age group (12–18 months of age), but effects may become apparent later throughout childhood. In infancy, executive functions are undifferentiated, or unrefined^(^ ^56^ ^)^. Over the course of development, as a function of experience-dependent neural specialisation, executive function differentiates into distinct constructs, namely working memory, cognitive flexibility and inhibitory control^(^ ^56^ ^,^ ^57^ ^)^. In addition, executive function differentiates from other cognitive functions, such as episodic memory^(^ ^58^ ^)^. Therefore, as cognitive functions differentiate, the effects of a nutrition intervention may become apparent on unique constructs. Executive function and memory are important predictors of school readiness and academic achievement^(^ ^58^ ^–^ ^60^ ^)^, even more so than intelligence quotient (IQ). As such, greater understanding of their response to nutrition interventions in early life should be prioritised for further research.

Our findings indicate that the intervention had a stronger impact on motor and mental development of children with a higher level of stimulation at baseline, suggesting that a minimum level of stimulation and resources at home are required before MNP interventions can provide benefits to development. These findings are consistent with the synergistic effect of stimulation and nutrition in early childhood. For instance, an effectiveness trial of iodised salt in Ethiopia found benefits of the intervention on mental development of children if their mothers had attended school^(^ ^61^ ^)^. Another study in Mexico resulted in larger impact of a group-based parenting programme on language development in children of mothers with any formal education compared with no education^(^ ^62^ ^)^. This threshold hypothesis is also supported by the literature showing larger gains in language and literacy outcomes from increases in quality of instruction for children from higher-quality classrooms compared with those from lower-quality classrooms^(^ ^63^ ^)^. However, these findings should be taken as preliminary given that the study design did not allow for follow-up of the same children and baseline values for stimulation were imputed.

Strengths of the study included its large sample size and cluster-randomised design, which allowed comparison across intervention and control communities. Clustering resulted in little contamination across groups. The use and validation of the tools to assess infant and young child development in rural northern India is an important addition to the literature. The DMC-II can be administered by trained non-specialists, and is a simple and quick measure of global development. The A-not-B and Elicited Imitation tasks require specialised training, but performed well in this context. Limitations of the study include the two cross-sectional surveys from baseline to endline rather than a follow-up of the same children. A longitudinal sample would have revealed differences in development outcomes on the same children and reduced confounding by having every child serve as their own control. However, the benefits of the design used, over following up the same children at two time points, are that every eligible child in the community received the intervention and no children were regarded as a special sample and given attention that would not model an effectiveness study. The timing of funding for the additional executive function and memory outcomes prevented us from measuring these cognitive functions at baseline. The lack of baseline measures precluded a difference-in-difference analysis. Some differences existed between intervention groups at baseline; however, these were accounted for in the statistical analyses. Mothers were not blinded to the intervention, and responses to the parent report DMC-II could have been biased by their expectations of the MNP. However, data collectors were blinded to the intervention group and would not have provoked a biased response.

The study’s significant impact on child development is important to inform the IFHI and other state-level nutrition and early child development initiatives. The impact we observed on motor and mental development from the intervention is equivalent to 10 and 16 d of development, respectively, when we compare the observed estimates of the effects with the change in development scores by child age in months. Despite the moderate coverage of the intervention, these achievements are important in the context of a state with extremely poor nutritional indicators and extreme poverty. Further, the threshold effect we see from stimulation indicates that programmes addressing both stimulation and nutrition could have important implications on early child development.

## Supplementary material

For supplementary material accompanying this paper visit http://dx.doi.org/10.1017/S000711451800140X. click here to view supplementary material
