# Supplementary material for: Effectiveness of a home fortification programme with multiple micronutrients on infant and young child development: a cluster-randomised trial in rural Bihar, India
Source: Br J Nutr. 2018 Jul 28;120(2):176–87. doi: 10.1017/S000711451800140X (PMC6088539; doi:10.1017/S000711451800140X)
Supplement: Supplementary file 1 [file S000711451800140Xsup001.docx]

Supplemental Table 1: Description of A-not-B and Elicited Imitation tests

| **Test** | **Description** |
| --- | --- |
| A-not-B | Research assistants worked in pairs; one interacted with the child while the other took notes and scored the measure. In the A-not-B task, the child is shown a desirable object hidden under a cloth (location A), within the child’s reach. After a brief delay, the child is allowed to search for and find the object. After several successes finding the object at a particular location, the object is then hidden under a cloth at an alternate location (B). Here, correct performance depends on the child’s ability to update their memory of the hiding place as well as to inhibit the response of searching at the location where the object was previously found. Children were asked to sit on the caregiver’s lap, equidistant from the cloths. Immediately after the toy was placed under a cloth, the delay period began. Children were scored as making an error if they reached to the empty cloth, if they did not reach at all over the course of 30 seconds, or if they reached simultaneously to both cloths. Initial side of hiding (left or right) was counterbalanced across children and visits, and side of hiding was reversed after every two consecutive successes. The first reversal trial had no delay. No reversal trial was administered until the child reached correctly on the two trials prior to the reversal. Each child was given four trial attempts to retrieve the object successfully under a given delay. If the child was successful in retrieving the object on two consecutive trials, the side of hiding was changed and the delay incremented by 3 seconds. This was continued until the child failed to retrieve the object on two consecutive trials or the maximum of 12 seconds delay was successfully passed (delays: 0, 3, 6, 9, 12 seconds) (Supplemental Figure 1). |
| Elicited Imitation | In the Elicited Imitation task, which measures episodic memory, the child was tested for immediate and delayed recall of two 2-step tasks and two 3-step sequences of action. For each sequence, the child was allowed to manipulate the objects for 30 seconds. Any actions completed during this time were recorded as “baseline”. Then, the research assistant, sitting in front of the child, modeled and narrated the sequence of actions in succession two times. They then returned the props to the child and invited them to imitate the exact sequence. The trial ended if the child pushed the props away or engaged in repetitive exploratory behaviors (e.g., banging the objects on the ground). The child was given two attempts to complete each sequence, if the first attempt was incomplete. Each child was tested on one 2-sequence task and one 3-sequence task immediately after modeling and one 2-sequence task and one 3-sequence task after a delay of 10 minutes (different tasks to those used for immediate recall). Ten minutes is long enough to reveal a deficit in individuals with compromised memory function due to medial-temporal lobe damage, and thus it is especially sensitive to the developmental integrity of the hippocampus. During the delay, the examiner filled the time by conducting the immediate imitation tests. The 2-sequence tasks were 1) lift a stand and attach a foam animal to the stand, and 2) mount a slide and roll a car down the slide. The 3-sequence tasks were 1) place a ball in a cup, cover it with a top, and shake it, and 2) pivot a stand, hang a bell on the stand, and hit the bell with a mallet. To score the children’s behavior, for each sequence, we calculated a total number of individual target actions produced (maximum=2 for 2-sequence tasks, maximum=3 for 3-sequence tasks) and the total number of pairs of actions produced in the target order (maximum=1 for 2-sequence tasks, maximum=2 for 3-sequence tasks). Only the first occurrence of each target action was considered so as to reduce credit that might be received due to chance or trial and error. |

Supplemental Table 2: Effect size for effect of MNP vs control for children from low compared to high stimulation households and mean DMC-II scores for children in MNP intervention group at endline by consumption of Jeevan Jyoti in the past one month^1^

|  | **Low stimulation at baseline^2^** | **High stimulation at baseline^2^** |  | **<10 sachets/month^3^** |  | **>= 10 sachets/month^3^** |  |  |
| --- | --- | --- | --- | --- | --- | --- | --- | --- |
|  | **Effect size (95% CI)** | **Effect size (95% CI)** | **P-value** | **Mean** | **SD** | **Mean** | **SD** | **P-value** |
| Motor development (out of 32) | 0.09 (-0.04, 0.22) | 0.20 (0.05, 0.34) | 0.004 | 18.4 | 6.7 | 20.0 | 5.8 | <0.001 |
| Mental development (out of 50) | 0.14 (0.01, 0.27) | 0.22 (0.08, 0.37) | <0.001 | 25.6 | 7.8 | 28.1 | 6.3 | <0.001 |
|  |  |  |  |  |  |  |  |  |
| Gross motor (out of 22) | 0.14 (0.01, 0.26) | 0.21 (0.06, 0.35) | 0.112 | 11.9 | 5.0 | 13.0 | 4.5 | <0.001 |
| Fine motor (out of 10) | -0.04 (-0.17, 0.10) | 0.13 (-0.01, 0.27) | <0.001 | 6.5 | 2.1 | 7.1 | 1.8 | <0.001 |
| Language (out of 15) | 0.15 (0.03, 0.28) | 0.22 (0.07, 0.36) | <0.001 | 5.2 | 2.7 | 5.9 | 2.4 | <0.001 |
| Personal-social (out of 28) | 0.11 (-0.02, 0.25) | 0.20 (0.06, 0.34) | <0.001 | 16.2 | 4.2 | 17.5 | 3.5 | <0.001 |
| Cognitive (out of 7) | 0.06 (-0.08, 0.19) | 0.10 (-0.04, 0.24) | <0.001 | 4.2 | 1.8 | 4.7 | 1.4 | <0.001 |
| ^1^Accounting for clustering by Health Sub-Center and nested Anganwadi Center. Analysis is adjusted for age of child, baseline hemoglobin, baseline home stimulation score, wealth index, maternal education, caste, and young mother. P-values are for interaction term. ^2^Scores were divided by low and high stimulation based on below or above median baseline value (≤5 vs >5). Low home stimulation N=2025; high home stimulation N=2273. ^3^Analysis includes only children at endline from the intervention group. Fewer than 10 sachets/month N=1643; more than or equal to 10 sachets/month N=531. MNP, multiple micronutrient powder; SD, standard deviation. | | | | | | | | |

Supplemental Figure 1: A-not-B task sequence

Toy hidden under cloth A

Child allowed to retrieve toy (no delay)

Child allowed to retrieve toy (no delay)

*Failure*

Stopped after four trial attempts

*Success on 2 consecutive trials*

*Failure*

Toy hidden under alternate cloth A/B

*Increment delay by 3 seconds*

Toy hidden under alternate cloth B

Stopped after four trial attempts

*Success on 2 consecutive trials*

*Etc.*

*Increment delay until a maximum of 12 second delay*

Child allowed to retrieve toy (3 second delay)

Supplemental Figure 2: Change in motor development scores from baseline to endline in intervention and control groups^1^

^1^ Point estimates are mean scores adjusted for clustering by Health Sub-Center and nested Anganwadi center and age of child, baseline hemoglobin, baseline home stimulation score, wealth index, maternal education, caste, and young mother. Error bars represent standard errors.

Supplemental Figure 3: Change in mental development scores from baseline to endline in intervention and control groups^1^

^1^ Point estimates are mean scores adjusted for clustering by Health Sub-Center and nested Anganwadi center and age of child, baseline hemoglobin, baseline home stimulation score, wealth index, maternal education, caste, and young mother. Error bars represent standard errors.
